# Supplementation of amylase combined with glucoamylase or protease changes intestinal microbiota diversity and benefits for broilers fed a diet of newly harvested corn

**DOI:** 10.1186/s40104-018-0238-0

**Published:** 2018-03-12

**Authors:** Dafei Yin, Xiaonan Yin, Xingyu Wang, Zhao Lei, Maofei Wang, Yuming Guo, Samuel E. Aggrey, Wei Nie, Jianmin Yuan

**Affiliations:** 1 State key Laboratory of Animal Nutrition, College of Animal Science and Technology, China Agricultural University, Beijing, China; 2 NutriGenomics Laboratory, Department of Poultry Science, University of Georgia, Athens, GA USA

**Keywords:** Amylase, Broiler, Glucoamylase, Microbiota diversity, Newly harvested corn, Protease

## Abstract

**Background:**

The effect of amylases combined with exogenous carbohydrase and protease in a newly harvested corn diet on starch digestibility, intestine health and cecal microbiota was investigated in broiler chickens.

**Methods:**

Two hunderd and eighty-eight 5-day-old female chickens were randomly divided into six treatments: a newly harvested corn-soybean meal diet (control); control supplemented with 1,500 U/g α-amylase (Enzyme A); Enzyme A + 300 U/g amylopectase + 20,000 U/g glucoamylase (Enzyme B); Enzyme B + protease 10,000 U/g (Enzyme C); Enzyme C + xylanase 15,000 U/g (Enzyme D); and Enzyme D + cellulase 200 U/g + pectinase 1,000 U/g (Enzyme E). Growth performance, starch digestibility, digestive organ morphology, and intestinal microbiota were evaluated in the birds at 16 and 23 d of age.

**Results:**

Compared with the control diet, supplementation with Enzyme A significantly decreased ileum lesion scoring at 16 d of age (*P* < 0.05); supplementation with Enzyme B or Enzyme C showed positive effects on ileal amylopectin and total starch digestibility (*P* < 0.05); Broilers fed with a diet supplemented with Enzyme D had a tendency to decrease body weight gain at 23 d. Enzyme E supplementation improved lesion scoring of jejunum and ileum at 16 d (*P* < 0.05), and increased ileal amylopectin or total starch digestibility at 23 d (*P* < 0.05). Supplementation of enzymes changed cecal microbiota diversity. High numbers of *Campylobacter, Helicobacter* and *Butyricicoccus, Anaerostipes and Bifidobacterium, Sutterella* and *Odoribacter* were the main genera detected in supplementations with Enzymes B, C, D, and E respectively.

**Conclusions:**

Supplementation with amylase combined with glucoamylase or protease showed a beneficial effect on starch digestibility and intestinal microbiota diversity, and increased growth of broilers fed with newly harvested corn.

**Electronic supplementary material:**

The online version of this article (10.1186/s40104-018-0238-0) contains supplementary material, which is available to authorized users.

## Background

In China, the production of corn is insufficient to meet the requirements for animal production, so a large amount of newly harvested corn is directly processed as animal feed each autumn. However, this material often causes rapid feed passage, incomplete grain digestion in the small intestine, and reduced performance in livestock.

The proportion of the components of plant feedstuffs for which there is no corresponding endogenous digestive secretions limits the nutrient and energy digestibility by poultry [1]. Cornstarch is composed of amylose (AM) and amylopectin (AP). Amylopectin makes up 70–80% of most starch sources. Amylopectin not only contains α-1, 4 -glucosidic bonds, but also α-1, 6-glucosidic bonds. In animals, only α-amylase can degrade α-1, 4 -glucosidic bonds, and amylopectase is required to hydrolyze amylopectin [1].

Additionally, corn has a high concentration of insoluble non-starch polysaccharides (NSP) such as xylans and cellulose [2], which limit the ability of digestive enzymes to access and fully digest the starch and protein components enclosed within the plant cells [3]. A previous study showed that starch granules are different in size and composition depending on the type and variety of cereal as well as the age of the cells in the developing endosperm [4]. Corn has large amounts of native trypsin inhibitors, ranging between 0.56 and 1.87 mg/g dry matter (DM) [5], which imposes restrictions on enzyme access to substrates, and is associated with high digesta viscosity in broiler chickens [6]. Slower digesta passage rate and impaired nutrient digestion, as well as greatly increased water intake provides greater opportunities for growth of *Clostridium perfringens* in the upper gut of broiler chickens [7, 8], which has a negative influence on gut health [9].

Supplementation with α-amylase shows a positive effect on digestibility of starch and organic matter, AME (apparent metabolizable energy) of diet, as well as feed conversion [10, 11]. However, no improvement in starch digestibility was observed by Mahagna [12] and Kaczmarek, [13] when α-amylase alone was supplemented to feed for broiler chicks during the first 14 d of age, and no beneficial effect on performance was observed by Yegani and Korver [14] and Kaczmarek [13].

Supplementation with appropriate NSP-degrading enzymes in new season grains diets could help hydrolyze NSP, reduce digesta viscosity and improve nutrient digestibility, thus increasing body-weight gain and feed conversion of broilers fed a corn-based diet [15]. Xylanase may increase the access of cell contents in feed to endogenous digestive enzymes due to hydrolysis of cell wall arabinoxylans and thus reduce the anti-nutritional effect of some polysaccharides [2, 16, 17]. Moreover, mixed enzyme preparations can effectively degrade indigestible cell constituents [18] or a large array of feed ingredients [19]. Supplementation with amylase and xylanase increases starch digestibility in the jejunum and ileum, AME and growth performance in broilers [1].

The gastrointestinal tract is populated with diverse assemblages of microbiota that play critical roles in the overall well-being of an animal [20]. The diversity and abundance of gut microbiota are affected by the dietary composition as well as by its digestibility [2, 20, 21]. NSPase supplementation improves digesta passage and nutrient digestion rate, leading to fewer opportunities for interaction between pathogens and substrates [22], and reducing levels of *Clostridium perfringens* [7]. Traditional identification methods for bacteria have limitations, wherase 16S rDNA analysis can identify all species present in a habitat including those that cannot be cultivated by conventional techniques [23]. 16S rRNA gene sequencing has been employed in a range of studies to assess the diversity and phylogenetic relationships of gut microbes and this has been proven to be a powerful tool for understanding the factors that shape microbial communities due to its informative and predictive potential [24]. Supplementation with NSPase can alter microbial profiles, as detected using Miseq Illumina sequencing [19].

Although much research has studied the effect of multienzyme preparations in corn/wheat-soy bean (SBM) diets, little is known of multienzyme preparations in newly harvested corn-SBM diets. The purpose of this experimental design was to use feed supplemented with amylase and combined with exogenous carbohydrases and/or protease to study the effect of different enzyme cocktails on the performance, nutrient utilization, hormone secretion, gut health and microbial profiles in broiler chickens.

## Methods

The present study was approved by China Agricultural University and conducted in accordance with the Guidelines for Experimental Animals.

### Enzymes

The enzymes were all purchased from Beijing Huameiyuan Biotechnology Co., LTD (Beijing). Alpha-amylase (*Aspergillus oryzae*) 3,000 U/g, amylopectase (*Bacillus amyloliquofaciens*) 2,000 U/g, glucoamylase (*Aspergillus usamil*) 200,000 U/g, protease (*Bacillus subtilis*) 800,000 U/g, xylanase (*Trichoderma longibrachiatum*) 240,000 U/g, cellulase (*Trichoderma harziamum*) 10,000 U/g, and pectinase (*Aspergillus niger)* 30,000 U/g (according to the manufacturer’s declaration).

### Birds and treatment diets

One-day-old Cobb 500 female-chicks were purchased from a commercial hatchery. Birds were raised in battery cages, fed ad libitum with commercial broiler starter diet without enzyme for the first 5 d. At 5 d, the chickens were individually weighed, and 288 chicks with similar body weight were then randomly allotted to six dietary treatments (six replicates per diet with eight chicks). The basal diet (control) composition and nutrient content is provided in Table 1. The corn used in the diet was newly harvested Zhengdan 958, sun dried for 1 wk post-harvest and contained about 14% moisture. The other five treatments were: Enzyme A (1,500 U/g α-amylase); Enzyme B (Enzyme A + 300 U/g amylopectase+ 20,000 U/g glucoamylase); Enzyme C (Enzyme B + protease 10,000 U/g); Enzyme D (Enzyme C + 15,000 U/g xylanase); and Enzyme E (Enzyme D + 200 U/g cellulase + 1,000 U/g pectinase). The nutrient values met or exceeded the recommendations of NY/T33–2004 [25].

**Table 1:** Composition of basal diet

Ingredients	Inclusion rate		Nutrient content
Corn	51.60	AME, Mcal/kg	2.95
Soybean meal	36.20	CP, %	21.00
Corn gluten meal	5.00	Lys, %	1.20
Soy oil	2.50	Met, %	0.50
Calcium hydrophosphate	2.10	M + C	0.85
Limestone	1.00	Thr	0.85
Salt	0.30	Ca, %	1.00
Minerals Premix^a^	0.20	AP, %	0.44
Vitamin Premix^b^	0.03		
50% Choline chloride	0.20		
*DL*-Methionine, 98%	0.20		
*L*-Lysine HCl, 98%	0.14		
Butylated hydroxytoluene (33%)	0.03		
Titanium dioxide	0.50		

^a^Provided per kg diet: Cu (CuSO_4_·5H_2_O), 8 mg; Zn (ZnSO_4_·H_2_O), 75 mg; Fe (FeSO_4_·H_2_O), 80 mg; Mn (MnSO_4_·H_2_O), 100 mg; Se (Na_2_SeO_3_), 0.30 mg; I [Ca(IO_3_)_2_·H_2_O], 0.35 mg

^b^Provided per kg diet: vitamin A (as retinyl acetate), 12,500 U; cholecalciferol, 2500 U; vitamin E (as *DL*-α-tocopherol acetate), 18.75 mg; menadione, 2.65 mg; thiamine, 2.5 mg; riboflavin, 6.0 mg; pyridoxine, 4.9 mg; pantothenic acid, 12 mg; niacin, 50 mg; folic acid, 1.25 mg; biotin, 0.0325 mg; cobalamine, 0.025 mg

### Animal rearing conditions

All birds were housed in two tier cages (100 cm × 70 cm) in an environmentally controlled house. The room temperature was initially set at 33°C then gradually reduced according to the age of the birds until reaching 23 °C at 21 d. Birds were kept on a 23:1 Light/Dark regimen with free access to feed and water. At d 16 post-treatment, one bird per replicate was removed for sampling. Between d 16 and 23, diets in all treatments were supplemented with 0.5% TiO_2_ (an inert marker). Body weight and feed intake per cage were recorded weekly to determine growth and feed efficiency. On d 23 birds were killed by intracardial administration of sodium pentobarbital (50 mg/kg of body weight) into the wing vein. Immediately following euthanasia the abdominal cavity was opened, the pancreas, liver and small intestines were removed, and the organ weights relative to body weight (BW) were calculated.

### Histo-morphology of the intestinal mucosa

At d 23, the duodenum, jejunum, and ileum were located and opened lengthwise. The intestine was examined microscopically and scored for gross lesions following a 0 to 6 scoring system described by Keyburn et al. [26]. Approximately 1 cm from each segments of the intestine were cut and placed separately in a 10% formalin solution for further processing to examine histo-morphological changes in the intestinal mucosa. The light microscopy evaluation and measurement of intestinal mucosal villus height and crypt depth were performed on a Zeiss Axiophot microscope (Carl Zeiss, Oberkochen, Germany). Measurements of villus height and crypt depth were made on 10 villi at 100× and 200× magnifications with imaging software (Image Pro Plus, Version 4.1.0.9; Media Cybernetics, Silver Spring, MD).

### Microbiota analysis

Fresh cecal samples were collected and stored at − 80 °C for further analysis. DNA was extracted from 0.18 to 0.22 g of cecal digesta using a QIA amp DNAStool Mini Kit (Qiagen). The DNA was recovered with 30 mL of AE buffer (10 mmol/L Tris-Cl, 0.5 mmol/L EDTA, pH 9.0; Qiagen). The 16S ribosomal RNA (rRNA) gene was analyzed to evaluate the bacterial diversity using Illumina Miseq (Novogene Bioinformatics Technology Co., Ltd.).

### Pyrosequencing

The normalized concentration of purified genomic DNA was used as a template to analyzed microbial communities. PCR amplification was performed using Takara Ex-Taq polymerase (Takara Bio, Shiga, Japan). The V3-V4 region of the 16S rRNA gene was amplified using eubacterial primers (341F: ACTCCTACGGGAGGCAGCAG, 806R: GGACTACHVGGGTWTCTAAT). PCR reactions were performed by initial denaturation at 94 °C for 3 min and then 28 cycles of 94 °C for 30 s, 53 °C for 40 s and 72 °C for 1 min, followed by a final elongation step at 72 °C for 5 min. Amplicon libraries were separated by agarose gel electrophoresis and purified using a QIA quick Gel Extraction Kit (Qiagen, Valencia, CA, USA). The DNA libraries were sequenced on Illumina HiSeq 2500 PE250 platform (Illumina, San Diego, US) for paired-end reads of 300 bp. The 16S rRNA gene sequences determined in this study were deposited in the NCBI Sequence Read Archive (SRA) database.

### Data processing

Sequence reads were analyzed by the quantitative insights into microbial ecology (QIME) software package (Uparse v7.0.1001, http://drive5.com/uparse/). Briefly, the raw sequence reads were quality trimmed and demultiplexed. The sequence reads were then clustered into operational taxonomic units (OTUs) by de novo OTU picking at a 97% level of sequence similarity. The taxonomic assignments for each representative sequence were obtained using the uclust consensus taxonomic classifier using the GreenGenes 13_8 database.

The microbial diversity indices of the samples (alpha diversity) were determined using the abundance-based coverage estimator (ACE), Chao1, observed OTUs, Shannon, and Simpson methods. Principal component analysis (PCA) was performed at the phylum and genus level. The abundance of microbial taxa was expressed as a percentage of the total 16S rRNA gene sequences, and the differences between the treatments were compared.

### Digestibility of nutrients

At 23 d of age, three birds per replicate were randomly selected and killed by intracardial administration of 5% sodium pentobarbital. Digesta of proximal and distal of ileum were collected gently, mixed and then freeze-dried, ground and analyzed for total starch, amylopectin, amylose and TiO_2_. The TiO_2_ was performed by the spectrophotometic method AOAC (975.21). Starch was determined by the *α*-amylase glucosidase method (996.11), as described by the Association of Official Analytical Chemists (2000) [27]. Amylose (AM) and amylopectin (AP) were determined using the method of Lin et al. [28]. The apparent ileal digestibility of nutrient or energy was calculated as described by Edwards and Gillis [29].

### Blood biochemistry

At 16 d and 23 d of age, one bird per pen was selected. Blood samples were taken from the wing vein and centrifuged at 3,600×*g* for 10 min to obtain serum to determine glucose (GLU), triglyceride (TG), total cholesterol (TC) and total bile acid (TBA) by using commercial analytical kits, according to manufacturer’s recommendations (Jian Cheng Bioengineering Institute, Nanjing, China). The content of insulin (INS), proglucagon (PG), triiodothyronine (T_3_) and tetraiodothyronine (T_4_) was determined by radioimmunoassay at the Beijing Sino-UK Institute of Biological Technology (Beijing, China).

### Statistical analysis

The results were analyzed using PROC ANOVA and Duncan’s Multiple Range Test using SPSS 15.0 for Windows (SPSS Inc., Chicago, IL) [30]. Percentage values were arcsine-transformed before analysis. Differences among means with *P* ≤ 0.05 were accepted as statistically significant.

## Results

### Growth and feed efficiency

Supplemented enzymes did not affect body weight gain (BWG) over 5–16 d of age (Table 2). There was a tendency difference between treatments on BWG during 17–23 d of age (*P* < 0.1). Supplemented Enzyme B and Enzyme C improved BWG of broilers between 17 and 23 d of age compared with Enzyme A or Enzyme D (*P* ≤ 0.05). Supplemented enzymes did not affect the average feed intake or feed conversion ratio (FCR) of the broilers (*P* > 0.05).

**Table 2:** Effect of enzyme^1^ supplementation in poultry diet on performance^2^

Treatments	BWG, g		AFI, g		FCR	
	5-16 d	17-23 d	5-16 d	17-23 d	5-16 d	17-23 d
Control	295	272	447	843	1.52	1.51
Enzyme A	303	247	458	845	1.51	1.56
Enzyme B	313	280	464	897	1.48	1.51
Enzyme C	318	277	461	887	1.45	1.48
Enzyme D	310	242	456	850	1.47	1.55
Enzyme E	313	255	454	859	1.45	1.52
SEM	4.0	5.0	4.6	4.6	0.017	0.024
*P*-value	0.527	0.061	0.877	0.416	0.794	0.769

^1^Enzyme A (1,500 U/g α-amylase; Enzyme B (Enzyme A + 300 U/g amylopectase + 20,000 U/g glucoamylase); Enzyme C (Enzyme B + protease 10,000 U/g); Enzyme D (Enzyme C + 15,000 U/g xylanase); Enzyme E (Enzyme D + 200 U/g cellulase + 1,000 U/g pectinase)

^2^*ABW* Average body weight, *BWG* Body weight gain, *AFI* Average feed intake, *FCR* Feed conversion ratio

### Digestibility and serum metabolites

Supplementation of enzymes did not affect amylose and amylopectin digestibility in proximal ileum, but supplemented Enzymes B, C and E increased distal ileal amylopectin and total starch digestibility in broilers at 23 d of age (*P* ≤ 0.05) (Table 3). Supplementation with Enzymes B, C, D or E significantly (*P* ≤ 0.05) increased serum glucose at d 16 compared to the control (Table 4). Similarly, supplementation with Enzymes C, D or E significantly increased serum TC content of broilers at 16 d of age compared with the control (*P* ≤ 0.05). Supplemented Enzyme E significantly decreased serum TBA content compared with the control (*P* ≤ 0.05) of broilers at 16 d of age. However, at d 23, supplementation with Enzyme B or Enzyme D significantly decreased serum TBA content of broilers compared with the control (*P* ≤ 0.05). There were differences in hormonal changes among treatments at d 16, but not d 23. There was no pattern of change of serum INS with respect to the treatments. However, plasma PG was significantly reduced in all treatment groups compared with the control. Both T_3_ and T_4_ appeared to increase in the treatment groups compared to the control (Table 5).

**Table 3:** Effect of enzyme^1^ supplementation in poultry diet on starch digestibility

Treatments	Proximal ileum			Distal ileum		
	Amylose, %	Amylopectin, %	Total starch, %	Amylose, %	Amylopectin, %	Total starch, %
Control	95.64	93.38	93.82	97.86	93.89^c^	94.87^c^
Enzyme A	97.06	95.13	95.38	97.61	96.12^abc^	96.51^abc^
Enzyme B	97.47	97.09	97.20	97.80	97.08^ab^	97.29^ab^
Enzyme C	97.93	96.27	96.71	97.93	97.58^a^	97.67^a^
Enzyme D	96.33	94.71	94.74	97.29	94.12^bc^	94.94^bc^
Enzyme E	97.39	96.51	96.73	98.47	97.30^a^	97.60^a^
SEM	0.550	0.647	0.607	0.416	0.592	0.492
*P*-value	0.266	0.256	0.080	0.671	0.033	0.025

^1^Enzyme A (1,500 U/g α-amylase); Enzyme B (Enzyme A + 300 U/g amylopectase + 20,000 U/g glucoamylase); Enzyme C (Enzyme B + protease 10,000 U/g); Enzyme D (Enzyme C + 15,000 U/g xylanase); Enzyme E (Enzyme D + 200 U/ g cellulase + 1,000 U/g pectinase)

**Table 4:** Effect of enzyme^1^ supplementation in poultry diet on serum biochemical indices

Treatments	Glu, mmol/L		TG, mmol/L		TC, mmol/L		TBA, mmol/L	
	16 d	23 d	16 d	23 d	16 d	23 d	16 d	23 d
Control	11.69^c^	13.31	0.77	0.46	2.51^c^	2.85	4.45^a^	5.60^a^
Enzyme A	12.40^bc^	12.96	0.48	0.39	2.84^bc^	2.91	3.48^ab^	3.84^abc^
Enzyme B	12.63^ab^	12.22	0.52	0.44	2.86^bc^	2.71	4.05^a^	2.16^c^
Enzyme C	13.30^a^	12.28	0.55	0.48	3.24^ab^	2.81	2.75^ab^	5.03^ab^
Enzyme D	12.67^ab^	13.66	0.53	0.44	2.98^ab^	3.08	3.17^ab^	3.09^bc^
Enzyme E	12.88^ab^	12.56	0.54	0.39	3.34^a^	3.14	1.86^b^	3.70^abc^
SEM	0.132	0.173	0.033	0.014	0.070	0.081	0.246	0.350
*P*-value	0.010	0.080	0.123	0.327	0.004	0.664	0.033	0.047

^1^Enzyme A(1,500 U/g α-amylase); Enzyme B(Enzyme A + 300 U/g amylopectase + 20,000 U/g glucoamylase); Enzyme C (Enzyme B + protease 10,000 U/g); Enzyme D (Enzyme C + 15,000 U/g xylanase); Enzyme E (Enzyme D + 200 U/ g cellulase + 1,000 U/g pectinase)

*Glu* glucose, *TG* triglyceride *TC* total cholesterol, *TBA* total bile acid

**Table 5:** Effect of enzyme^1^ supplementation of in poultry diet on serum endocrine hormone levels

Treatments	INS, μIU/mL		PG, pg/mL		T_3_, ng/mL		T_4_, ng/mL	
	16 d	23 d	16 d	23 d	16 d	23 d	16 d	23 d
Control	12.75^bc^	11.55	121.52^a^	86.25	0.65^b^	0.69	18.47^b^	20.55
Enzyme A	13.21^b^	12.49	90.11^b^	78.60	0.80^ab^	0.68	19.54^b^	22.55
Enzyme B	15.55^a^	12.23	85.86^b^	84.84	0.97^a^	0.64	28.97^a^	24.52
Enzyme C	10.85^c^	12.69	90.14^b^	73.07	0.92^a^	0.68	22.36^b^	22.41
Enzyme D	13.08^b^	13.59	98.52^b^	79.97	0.84^ab^	0.72	19.05^b^	23.55
Enzyme E	12.79^bc^	11.77	88.89^b^	82.26	0.79^ab^	0.75	23.33^ab^	28.37
SEM	0.345	0.258	3.053	1.511	0.029	0.025	0.991	1.219
*P*-value	0.002	0.306	0.002	0.131	0.017	0.884	0.011	0.580

^1^Enzyme A(1,500 U/g α-amylase); Enzyme B(Enzyme A + 300 U/g amylopectase + 20,000 U/g glucoamylase); Enzyme C (Enzyme B + protease 10,000 U/g); Enzyme D (Enzyme C + 15,000 U/g xylanase); Enzyme E (Enzyme D + 200 U/ g cellulase + 1,000 U/g pectinase)

*INS* insulin, *PG* proglucagon, *T*_*3*_ triiodothyronine, *T*_*4*_ tetraiodothyronine

### Organ weight, lesion scores and intestine anatomy

There were no differences in liver and pancreas weights at 16 and 23 d of age with treatment, except for the significant difference between Enzymes B and E regarding pancreas weight at 23 d (Table 6). At 16 d of age, there were minor weight differences in duodenum, jejunum and ileum among treatments. However, the weights of the different sections of the intestines were all significantly heavier in the control group compared with the treatment groups. By d 23, there were no significant differences in the duodenum and ileum between the control and enzyme supplementation groups. The jejunal weight in the group supplemented with Enzyme E was significantly lighter than the control and all treatment groups except Enzyme A. Supplemented enzymes significantly affected lesion scoring of the intestine of broilers at 16 d but not 23 d of age (Table 7). Enzyme supplementation generally reduced intestinal lesion scores at d 16. Supplemented Enzyme D significantly decreased lesion scoring of the duodenum compared with the control (*P* ≤ 0.05). In the jejunum and ileum, supplementation with Enzymes C, D and E significantly reduced lesion scores compared with the control. Villi height was significantly higher in the enzyme-treated groups compared to the control at 16 d, but at 23 d the differences had disappeared (Table 8). Consequently, the ratio of villi height and crypt depth was significantly higher in the treatment groups compared with the control at d 16, but not at d 23.

**Table 6:** Effect of enzyme^1^ supplementation in poultry diet on the relative weight of digestive organs (%)

Treatments	d 16					d 23				
	Duodenum	Jejunum	Ileum	Liver	Pancreas	Duodenum	Jejunum	Ileum	Liver	Pancreas
Control	1.48^a^	2.60	1.93	1.08	0.45	1.27	2.22^ab^	1.82	2.31	0.40^ab^
Enzyme A	1.37^a^	2.18	1.73	1.13	0.41	1.10	1.92^cd^	1.33	2.31	0.35^bc^
Enzyme B	1.20^b^	2.29	1.83	1.19	0.42	1.12	2.35^a^	1.65	2.41	0.42^a^
Enzyme C	1.40^a^	2.30	1.57	1.03	0.40	1.14	2.06^abc^	1.66	2.42	0.37^abc^
Enzyme D	1.41^a^	2.14	1.65	1.14	0.41	1.21	2.05^bc^	1.67	2.46	0.35^bc^
Enzyme E	1.38^a^	2.19	1.56	1.13	0.40	1.09	1.73^d^	1.50	2.34	0.34^c^
SEM	0.021	0.050	0.047	0.024	0.010	0.030	0.049	0.057	0.037	0.009
*P*-value	0.001	0.072	0.077	0.542	0.848	0.488	0.001	0.180	0.805	0.033

^1^Enzyme A(1,500 U/g α-amylase); Enzyme B(Enzyme A + 300 U/g amylopectase + 20,000 U/g glucoamylase); Enzyme C (Enzyme B + protease 10,000 U/g); Enzyme D (Enzyme C + 15,000 U/g xylanase); Enzyme E (Enzyme D + 200 U/ g cellulase + 1,000 U/g pectinase), respectively

**Table 7:** Effect of enzyme^1^ supplementation in poultry diet on lesion scoring of the intestine

Treatments	d 16			d 23		
	Duodenum	Jejunum	Ileum	Duodenum	Jejunum	Ileum
Control	2.50^a^	2.60^a^	2.40^a^	1.17	2.00	1.67
Enzyme A	1.33^ab^	1.50^ab^	1.00^bc^	0.40	2.17	1.33
Enzyme B	1.50^ab^	1.50^ab^	1.67^ab^	1.00	1.83	1.83
Enzyme C	1.17^ab^	0.60^b^	0.33^c^	0.80	2.00	2.40
Enzyme D	0.67^b^	0.83^b^	0.67^bc^	1.20	2.00	1.60
Enzyme E	1.40^ab^	0.50^b^	1.00^bc^	1.00	2.17	1.83
SEM	0.197	0.224	0.258	0.174	0.213	0.207
*P*-value	0.033	0.023	0.005	0.838	0.998	0.828

^1^Enzyme A (1,500 U/g α-amylase); Enzyme B (Enzyme A + 300 U/g amylopectase + 20,000 U/g glucoamylase); Enzyme C (Enzyme B + protease 10,000 U/g); Enzyme D (Enzyme C + 15,000 U/g xylanase); Enzyme E (Enzyme D + 200 U/ g cellulase + 1,000 U/g pectinase)

**Table 8:** Effect of enzyme^1^ supplementation in poultry diet on intestinal histomorphology

Treatments	Duodenum						Jejunum		
	Villus height, μm		Crypt depth, μm		V/C		Villus height, μm	Crypt depth, μm	V/C
	16 d	23 d	16 d	23 d	16 d	23 d	23 d	23 d	23 d
Control	1189^a^	1542	280	241	4.42^a^	6.60^b^	943	194	5.43
Enzyme A	1774^b^	1822	296	257	6.43^b^	7.31^ab^	1050	207	5.29
Enzyme B	1717 ^b^	1648	254	267	7.08^b^	6.47^b^	953	211	4.93
Enzyme C	1736^b^	1768	268	250	7.06^b^	7.51^ab^	977	218	4.66
Enzyme D	1828^b^	1875	275	271	7.02^b^	7.39^ab^	880	195	4.82
Enzyme E	1894^b^	1816	257	212	7.68^b^	8.89^a^	1037	182	5.92
SEM	49.2	37.8	9.3	7.1	0.260	0.238	19	5.8	0.195
*P*-value	< 0.001	0.092	0.817	0.185	0.002	0.041	0.089	0.515	0.468

^1^Enzyme A (1,500 U/g α-amylase); Enzyme B (Enzyme A + 300 U/g amylopectase + 20,000 U/g glucoamylase); Enzyme C (Enzyme B + protease 10,000 U/g); Enzyme D (Enzyme C + 15,000 U/g xylanase); Enzyme E (Enzyme D + 200 U/ g cellulase + 1,000 U/g pectinase), respectively

### Microbial profile

A total of 1,364,610 sequence reads were recovered from a total of 24 samples from 6 treatments. Average Rarefaction curves indicated that a high coverage had been achieved in all samples (Additional file 1: Figure S1). The number of OTUs and sample richness and diversity are shown in Table 9. Good’s coverage index was almost constant (0.995~ 0.996) in the six treatments, which indicated a high coverage. The samples from the group supplemented with Enzyme E had lowest number (529) of OTUs while samples from the control group showed the highest number (595) of OTUs among treatments.

**Table 9:** The number of OTUs and sample richness and diversity of cecal microbiota

Treatments	OTUs per sample	Good’s coverage	Chao1 (richness)	Shannon (diversity)	Simpson (diversity)
Control	595	0.995	581.59	5.89	0.95
Enzyme A	568	0.995	631.27	5.78	0.96
Enzyme B	537	0.995	571.41	5.45	0.94
Enzyme C	555	0.996	586.81	5.72	0.95
Enzyme D	546	0.996	524.78	5.86	0.95
Enzyme E	529	0.996	492.43	5.64	0.94

Enzyme A (1,500 U/g α-amylase); Enzyme B (Enzyme A + 300 U/g amylopectase + 20,000 U/g glucoamylase); Enzyme C (Enzyme B + protease 10,000 U/g); Enzyme D (Enzyme C + 15,000 U/g xylanase); Enzyme E (Enzyme D + 200 U/ g cellulase + 1,000 U/g pectinase)

**Fig. 1:**
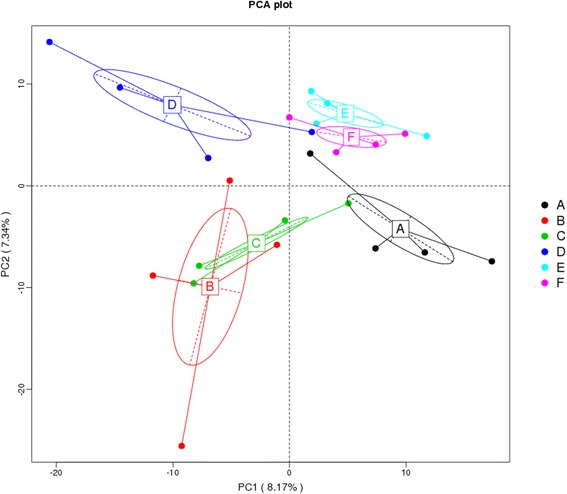
Principal component analysis (PCA) of microbiota community by Bray-Curtis distance. The circles were drawn around microbiota from the same treatment. Treatment information: (**a)**: control; (**b**): Enzyme A (1,500 U/g α-amylase; C: Enzyme B(Enzyme A + 300 U/g amylopectase + 20,000 U/g glucoamylase); D: Enzyme C (Enzyme B + 10,000 U/g protease); E: Enzyme D (Enzyme C + 15,000 U/g xylanase); F: Enzyme E (Enzyme D + 200 U/g cellulase + 1,000 U/g pectinase)

The richness of the bacterial community is reflected by Chao 1 index, which ranged from 492.43 to 631.27 among all treatments (Additional file 1:Figure S2 ). The Shannon and Simpson indices, reflecting the bacterial community diversity, ranged from 5.45 to 5.89 and 0.94 to 0.96, respectively (Additional file 1: Figure S2 ). However, based on those sequence metrics and sample richness and diversity parameters, there were no significant differences among all treatments. As shown in Fig. 1, the relationships among bacterial communities from different treatments were represented by principal component analysis (PCA). Based on Bray-Curtis dissimilarity, ANOSIM showed statistically significant differences in bacterial community among the treatments (*P* ≤ 0.05).

**Fig. 2:**
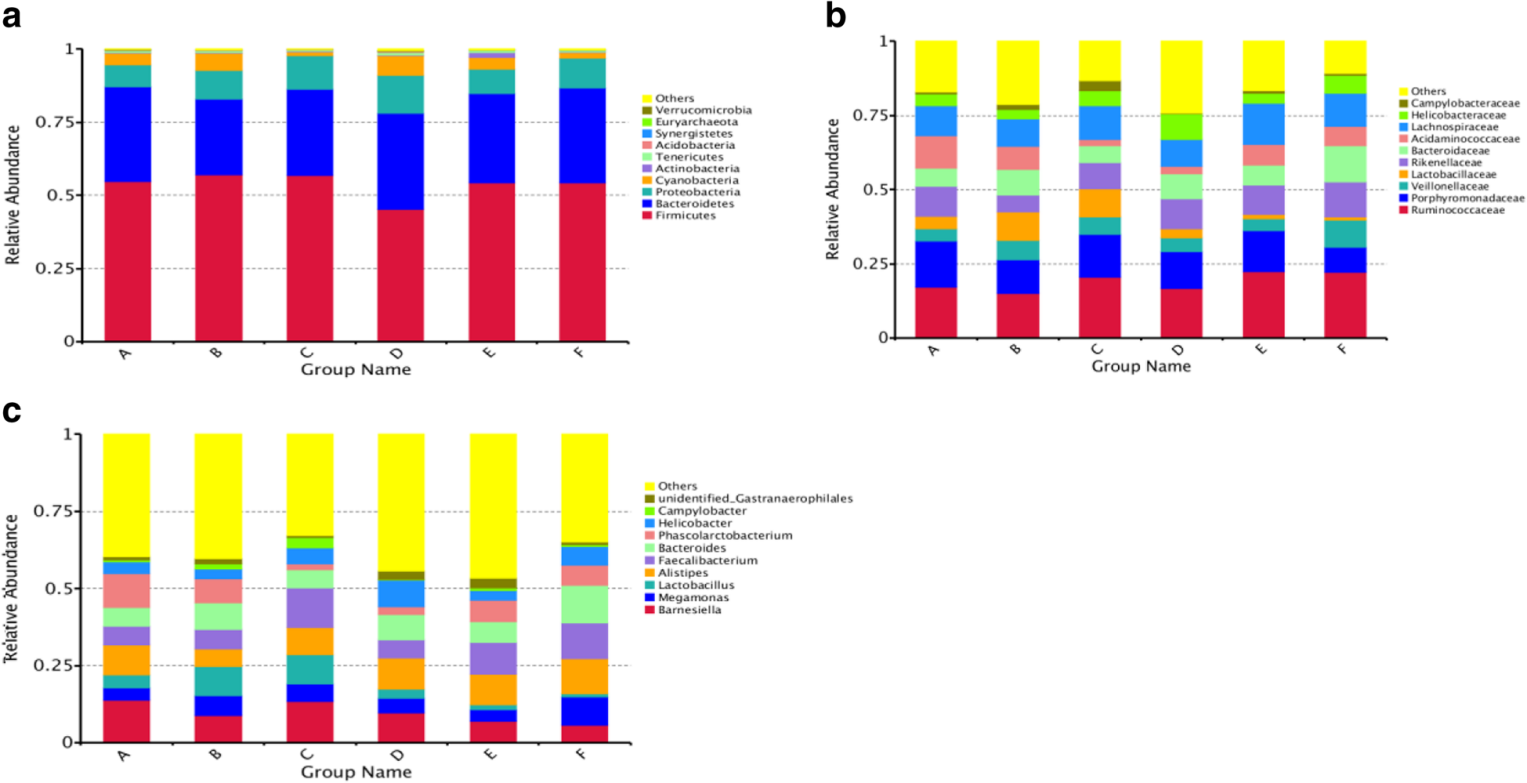
Relative abundance of the dominant bacterial communities in the cecal digesta of 23-day-old broiler chickens at phylum (**a**), family (**b**) and genus (**c**) level Each bar represents the relative abundance of each treatment. Each color represents a particular bacterial phylum. A: the control; B: Enzyme A (1,500 U/g α-amylase; C: Enzyme B(Enzyme A + 300 U/g amylopectase + 20,000 U/g glucoamylase); D: Enzyme C (Enzyme B+ 10,000 U/g protease); E: Enzyme D (Enzyme C + 15,000 U/g xylanase); F: Enzyme E (Enzyme D + 200 U/g cellulase + 1,000 U/g pectinase).

The most abundant phylum was Fimicutes, followed by Bacteroidetes (Fig. 2a). The relative abundances of Fimicutes and Bacteroidetes ranged from 45.24% to 60.28% and 25.98% to 32.90%, respectively (S1 Table). Those two dominant phyla dominated around 80% of the bacterial community. The remaining 20% of the bacterial population belonged to Proteobacteria, Cyanobacteria, Actinobacteria, Tenericutes, Acidobacteria, Synergistetes, Euryarchaeota, Verrucomicrobia and others (Fig. 2a and Additional file 1: Table S1). The number of OTUs classified as Acidobacteria phylum increased (*P* ≤ 0.05) in the group supplemented with Enzyme C compared with supplementation of Enzyme D or Enzyme E. Supplementation of Enzyme D significantly decreased the number of OTUs classified as Synergistetes phylum, while the relative abundance of Synergistetes phylum was increased in the control group (*P* ≤ 0.05). Most OTUs were classified within the families Ruminococcaceae and Porphyromonadaceae, followed by Lachnospiraceae, Veillonellaceae, Lactobacillaceae, Rikenellaceae, Bacteroidaceae and *Acidaminococcaceae* (Fig. 2b and Additional file 1: Table S2). The OTUs, belonging to the family Lactobacillaceae, were significantly enriched in the groups supplemented with Enzyme A or Enzyme B, and those OTUs were uniformly depleted in the presence of Enzyme D or Enzyme E. Adding Enzyme B or Enzyme C to diets decreased the number of OTUs classified as family Acidaminococcaceae when compared with the control group. Similarly, the abundance of *Lactobacillus*, belonging to Lactobacillaceae family (Additional file 1: Figure S3), was significantly increased with supplemented Enzyme A or Enzyme B while those bacteria were depleted in the groups with Enzyme D or Enzyme E (Fig. 2c and Additional file 1: Table S3). Meanwhile, the administration of Enzyme B or Enzyme C resulted in a decreased representation of the abundance of *Phascolarctobacterium* (Fig. 2c and Additional file 1: Table S3), which was most dominant in Acidaminococcaceae family (Additional file 1: Figure S3).

**Fig. 3:**
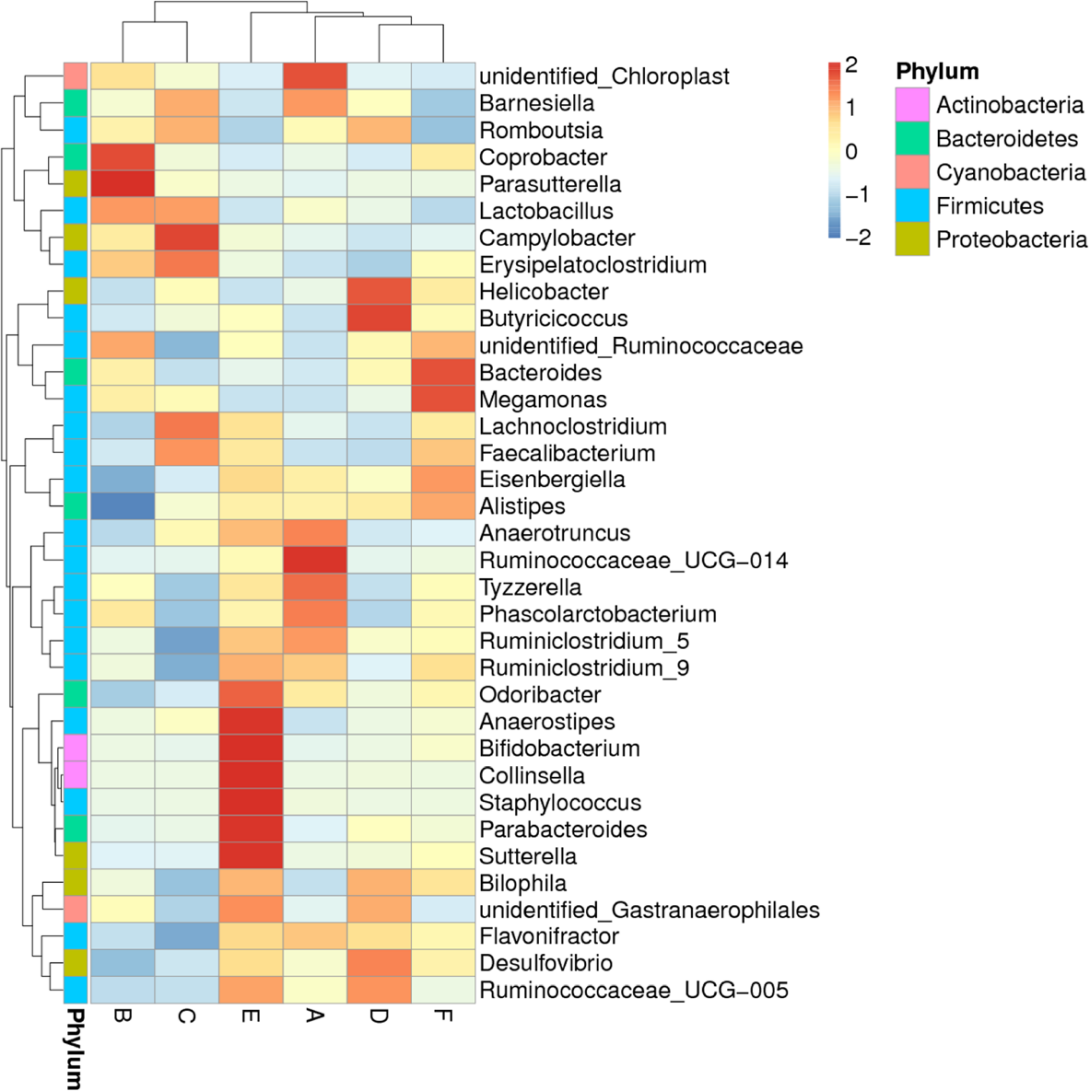
Hierarchically clustered heatmap analysis of the highly represented bacterial taxa (at the genus level) found in the cecal digesta communities of 23-day-old broiler chickens. The relative percentages (%) of the bacterial genus are indicated by varying color intensities according to the legend at the top of the figure. Bacterial genus names are listed on the right side of the heatmap, and the phylum names are listed on the left. Dendrograms at the top and left of the map indicate the phylogenetic relationship between the treatments and genus; the intensity of the cell color represents the abundance of the amplicons belonging to each genus. (**a**): the control; (**b**): Enzyme A (1,500 U/g α-amylase; (**c**): Enzyme B (Enzyme A + 300 U/g amylopectase + 20,000 U/g glucoamylase); (**d**): Enzyme C (Enzyme B+ 10,000 U/g protease); (**e**): Enzyme D (Enzyme C + 15,000 U/g xylanase); (**f**): Enzyme E (Enzyme D + 200 U/g cellulase + 1,000 U/g pectinase)

The heatmap in Fig. 3 shows the relative abundances of various bacteria at the genus level in the different treatments. The primary genera of bacteria in the control group were in the Ruminococcaceae, phylum Firmicutes, and included *Anaerotruncus, Tyzzerella* and *Phascolarctobacterium*. Additionally, the number of *Chloroplast* and *Barnesiella* also increased in the control group. Supplementation with Enzyme A shifted the bacterial community, and many *Coprobacter* and *Parasutterella* were detected. Many *Campylobacter* were associated with supplementation by Enzyme B. The primary genera were *Helicobacter* and *Butyricicoccus* in the group supplemented with Enzyme C. In contrast to other groups, the group supplemented with Enzyme D was enriched in *Anaerostipes*, *Bifidobacterium, Collinsella, Staphylococcus, Parabacteroides, Sutterella* and *Odoribacter*. In the group supplemented with Enzyme E, *Bacteroides* and *Megamonas* were the primary genera.

## Discussion

Corn (grain) contains approximately 9% NSP [31], and improved digestibility of NSP is expected to increase the nutrient availability. When the broiler diet was supplemented with only α-amylase, the total starch digestibility improved slightly, but the improvement was not significant. Based on the literature, data on diet supplementation with α-amylase are conflicting. Whereas Mahagna et al. [12], Ritz et al. [26] and Shapiro and Nir [28] observed no improvement in digestibility, Jiang et al. [11] observed significant improvements in performance due to α-amylase supplementation. Svihus [32] argues that specific diet properties, cereal type, inclusion level and bird related factors might contribute to the variation in the outcomes of supplementing poultry diets with α-amylase. Glucoamylase is expected to hydrolyze both α-1, 4- and α-1, 6-glucosidic linkage bonds, and amylopectase is expected to hydrolyze α-1, 6 bonds; thus, Enzyme B should be most likely to improve total starch digestion. In fact, Enzyme B slightly improved the digestibility of amylopectin in the proximal ileum. Because starch is composed of 70–80% amylopectin, a slight improvement in amylopectin digestion suggests that including a combination of amylopectase and α-amylase in poultry diets could improve starch digestibility compared with α-amylase alone. However, the inclusion of Enzyme B did not significantly change growth, feed intake or feed efficiency. We observed that Enzymes D and E decreased intestine and pancreas weights. Exogenous supplementation with certain enzymes could be antagonistic to secretion of some endogenous enzymes. Mahagna et al. [12] reported a similar observation.

The degree to which exogenous enzymes added to poultry diet may affect performance depends on the type and amount of cereal in the diet, the level of anti-nutritional factors in the diet, the spectrum and level of enzymes and the type of animal receiving the feed [33]. Choct et al. [7] found that corn contains protease and other inhibitors. A bi-functional Hageman factor inhibitor from corn inhibits endogenous protease and amylase activities [5, 34], demonstrating that the average native trypsin inhibitor activity (TIA) was 1.27 ± 0.33 mg/g DM and ranged between 0.56 and 1.87 mg/g DM in 12 corn kernel samples from Germany and Austria. Therefore, supplementing feed with proteases could improve the digestion of protein-based anti-nutrients. In the current study, protease was included in the Enzyme C supplementation, and this treatment significantly improved distal ileum starch digestibility compared with that in the control diet. Including proteases in the diet may change the starch-protein interface and affect digestibility [35]. Anti-nutrition factors are primary determinants of the availability of energy and other nutrients for poultry [6], which can form a viscous gel in the gut and affect the rates of digestion and absorption of nutrients, leading to diarrhea and reducing animal performance [36]. Although we did not study the rate of passage of digesta, we observed some stickiness among the fecal droppings in the control.

A large proportion of starch is entrapped in cell wall material, particularly from areas of the endosperm close to the aleurone layer [37]; therefore, including enzymes with the capability of digesting cell wall material should make starch more easily accessible to starch digesting enzymes. Xylanase may increase access to entrapped nutrient components by destroying some fractions of the plant cell walls of grains, allowing α-amylase access to starch fractions [38, 39]. Additional amylase and protease can effectively degrade indigestible cell constituents and increase the digestibility of nutrients in the broiler feed [18]. In previous studies, for birds fed corn-soybean diets supplemented with exogenous enzymes, nutrient digestibility increased [15, 40], nutritive variation of ingredients was eliminated and performance improved [41]. Munyaka et al. [19] showed that xylanase and β-glucanase increased starch digestibility in birds fed a wheat-based diet. Supplementation with amylase and xylanase increase starch digestibility in the jejunum and ileum by 3.5% and 2.4%, respectively, leading to improvements in growth performance, energy and starch digestibility in broilers [1]. However, in the current study, supplementation with NSP-degrading enzymes D (xylanase) and E (pectinase) did not benefit performance, although Enzyme E marginally increased digestibility of amylopectin and total starch. Others show that the effects of enzymes may not always be beneficial and that some low-molecular-weight NSP hydrolysis products may adversely affect protein digestion by chickens. A multi-carbohydrase enzyme not only hydrolyzed water-soluble NSP but also partially depolymerized the water-insoluble NSP fraction of feed ingredients, which significantly increased water-soluble and decreased water-insoluble NSP concentrations in the ileal digesta of birds fed enzyme-supplemented diets [16]. This result may explain why no significant benefits were derived from supplementing diets with multienzymes in the current study.

Nutritional factors affect intermediary metabolism, resulting in changes in serum metabolite levels in poultry [42]. Blood GLU concentrations affect serum total cholesterol and triglyceride concentrations [43], and plasma GLU levels reflect the rate of starch digestion [44]. We observed that supplementing the diet with Enzymes B, C, D or E increased serum GLU and TC at d 16. Supplementing diets with enzymes has the potential to change digesta properties and release more simple sugars, amino acids and di-peptides, increasing their metabolic availability. A previous study showed that NSP could affect bile salts and decrease the digestibility of oil [45]. However, no clear pattern was observed for the level of TBA with supplementation of poultry diets with exogenous enzymes. A close relationship between the somatotropic and thyrotropic axes in the regulation of growth and development of broiler chickens plays an important role in poultry growth [46]. Nutritional status is an important factor in the regulation of blood hormones and intermediary metabolism in broiler chickens [47]. The addition of dietary enzymes increases the digestion of feed and the absorption of nutrients, which affect hormone concentrations. Glucose is the primary trigger for the postprandial changes in metabolic and endocrine functioning [47]. Insulin levels may affect growth and nutrient utilization [48]. Gao et al. [49] reported that increases in nutrient digestion and absorption resulting from enzyme supplementation affected hormonal levels. We observed that Enzyme B increased the INS level, whereas Enzyme C affected T_3_ and T_4_ levels. Other studies show a relationship between T_3_ and growth [50]; however, we did not observe any growth trends associated with levels of T_3_ and T_4_ in the current study. Enzyme supplementation changed the villi height:crypt depth ratio, similar to the observations of Ritz et al. [51] and Onderci et al. [10]. Changes in intestinal anatomy can affect lesion scores and morphology. We observed that lesion scores were reduced at d 16 with enzyme supplementation. Gut anatomy and morphology invariably change the surface area of the gut and improve digestibility of NSP, and as a consequence, a reduction in digesta viscosity is expected. Substrate modification in the gut by enzyme supplementation can also change the dynamics of intestinal microbes relying on these substrates. Wagner and Thomas [52] demonstrated that the growth dynamics of over 600 species of organisms harbored in the gut respond to changes in the diet.

Gut microbiota perform diverse functions and participate in carbohydrate metabolism, fiber degradation, and immune maintenance; however, the functions may be influenced by diet, genotype, feeding pattern and inflammation [53]. Thus, changes in the intestinal environment can affect the microbial population dynamics in the gut and thereby influence functions. According to Torok et al. [54], digesta flow rate, pH, anoxic conditions, types of endogenous and dietary substrates, inhibitory factors such as bacteriocins and short-chain fatty acids (SCFA), and competition influence gut microflora. For example, incomplete digestion of starch in the small intestine favored growth of *Clostridium perfringens* in the upper gut [8], and resistant starch is also associated with increased numbers of *Brachyspira hyodysenteriae* and increased incidence of clinical dysentery in swine [55]. Supplementation of diets with exogenous enzymes can alter the microbial profile [56]. In the current study, the most abundant phylum was Fimicutes, followed by Bacteroidetes (Fig. 2a), and those two dominant phyla composed approximately 80% of the entire bacterial community. The other approximately 20% of bacteria were in the phyla Proteobacteria, Cyanobacteria, Actinobacteria, Tenericutes, Acidobacteria, Synergistetes, Euryarchaeota, Verrucomicrobia and others (Fig. 2a and Additional file 1: Table S1), as also reported by Oakley et al. [57] and Neumann and Suen [58].

The PCA axis 1 accounted for 8.17% of the intertreatment variation, and the axis 2 accounted for 7.34% of the intersample variation. Although the intertreatment variability explained was small, clear differences were observed in the gut microbiota populations among the treatments. In the current study, enzyme supplementation altered the relative abundance of cecal microbiota at the phylum and genus levels. The degradation products of the different treatments were expected to be different, with different enzyme combinations causing different changes in the substrate composition and digesta viscosity. In the current study, the number of OTUs classified as *Campylobacter, Helicobacter* and *Megamonas* was apparently enriched in treatments supplemented with Enzymes B, C and E, respectively. Sergeant et al. [59] suggested that these enzymes might improve uptake of hydrogenases and consequently be beneficial to both the bacterial community and to energy absorption.

With changes in the diet, some microbes can grow opportunistically to high levels or have growth suppressed to low levels, which in each case, can lead to certain maladies [60]. The number of OTUs and diversity apparently declined with enzyme supplementation, particularly in the treatment with Enzyme E. The diversity of gut microbiota may be associated with the proportion of indigestible components in the diet. *Megamonas* contains several genes that encode a series of polysaccharide degradation enzymes, such as cellobiose phosphotransferase and endo-glucanases, which aid in degrading complex polysaccharides to cellobiose and subsequent digestion to glucose [59]. Similarly, *Megamonas* and *Bacteroides* were also enriched in the treatment with Enzyme E. *Bacteroides* can secrete enzymes that participate in cellobiose and xylan degradation and that are also involved in polysaccharide utilization systems [59, 61] and short-chain fatty acid metabolism [60]. *Bacteroides* are indole negative, resistant to 20% bile, and produce acetic acids and a smaller amount of succinic acids as the primary end products of glucose metabolism [62]. Vahjen et al. [63] demonstrated that exogenous supplementation with xylanase led to increased counts of lactobacilli, which was a result also confirmed in a study by Nian et al. [64] in which, simultaneously, coliform and *Salmonella* were reduced. However, in the current study, coliform and *Salmonella* were not members of the enriched populations. *Bacteroides* may deconjugate bile acids, which can lead to poor fat absorption and storage [65]. Thus, the relationship between *Bacteroides* and the level of TBA should be explored further. For *Megamonas*, Scupham et al. [66] presented some data that suggest this genus is antagonistic to *Campylobacter*. Apparently, changes in gut microbial diversity may distort homeostasis, which affects the immune state of the host [53].

## Conclusions

It was concluded from the present study that a newly harvested corn diet of broilers requires supplementation with carbohydrase and protease to increase starch digestibility and increase the levels of INS and T_3_ to improve broiler body weight. Supplementation with dietary exogenous enzymes modulates the relative abundance of specific bacteria. Increases in population levels of polysaccharide-producing bacteria and decreases in destructive bacteria in chickens may be an intestinal health-promoting attribute and may contribute to starch utilization. Amylase combined with glucoamylase or protease is recommended for broiler diets with newly harvested corn.

## Additional file

Additional file 1: **Table S1.** Relative abundance of the dominant bacterial in the cecal digesta communities of 23-day-old broiler chickens at phylum level. **Table S2.** Relative abundance of the dominant bacterial community in the cecal digesta of 23-day-old broiler chickens at family level. **Table S3.** Relative abundance of the dominant bacterial community in the cecal digesta of 23-day-old broiler chickens at genus level. **Figure S1.** Rarefaction curves of samples clustered at 95% sequences identity. **Figure S2.** Richness and diversity of microbiome in cecal content. Chao 1 (a), Shannon diversity (b) and Simpson (c) indices are represented by box plots. Figure S3 Detailed taxon tree (DOCX 403 kb)

## Abbreviations

ABW: Average body weight
ACE: Abundance-based coverage estimator
AFI: Average feed intake
AM: Amylose
AP: Amylopectin
BWG: Body weight gain
DM: Dry matter
FCR: Feed conversion ratio
GLU: Glucose
INS: Insulin
NSP: Non-starch polysaccharides
OUTs: Operational taxonomic units
PCA: Principal component analysis
PG: Proglucagon
SBM: Soybean meal
SCFA: Short chain fatty acid
T_3_: Triiodothyronine
T_4_: Tetraiodothyronine
TBA: Total bile aid
TC: Total cholesterol
TG: Triglyceride
TIA: Trypsin inhibitor activity
