# Supplementary material for: Supplementation of amylase combined with glucoamylase or protease changes intestinal microbiota diversity and benefits for broilers fed a diet of newly harvested corn
Source: J Anim Sci Biotechnol. 2018 Mar 12;9:24. doi: 10.1186/s40104-018-0238-0 (PMC5846306; doi:10.1186/s40104-018-0238-0)
Supplement: Supplementary file 1 — Table S1. Relative abundance of the dominant bacterial in the cecal digesta communities of 23-day-old broiler chickens at phylum level. Table S2. Relative abundance of the dominant bacterial community in the cecal digesta of 23-day-old broiler chickens at family level. Table S3. Relative abundance of the dominant bacterial community in the cecal digesta of 23-day-old broiler chickens at genus level. Figure S1. Rarefaction curves of samples clustered at 95% sequences identity. Figure S2. Richness and diversity of microbiome in cecal content. Chao 1 (a), Shannon diversity (b) and Simpson (c) indices are represented by box plots. Figure S3 Detailed taxon tree (DOCX 403 kb) [file 40104_2018_238_MOESM1_ESM.docx]

**Supporting Information**

**S1 Table. Relative abundance of the dominant bacterial in the cecal digesta communities of 23-day-old broiler chickens at phylum level**

|  | Firmicutes | Bacteroidetes | Proteobacteria | Cyanobacteria | Actinobacteria | Tenericutes | Acidobacteria | Synergistetes | Euryarchaeota | Verrucomicrobia | Others |
| --- | --- | --- | --- | --- | --- | --- | --- | --- | --- | --- | --- |
| Control | 54.70 | 32.44 | 7.48 | 4.02 | 0.134 | 0.717 | 0.128^bc^ | 0.025^a^ | 0.000 | 0.0069^b^ | 0.194^b^ |
| Enzyme A | 57.02 | 25.98 | 9.71 | 5.96 | 0.134 | 0.259 | 0.325^ab^ | 0.016^ab^ | 0.000 | 0.0315^a^ | 0.271^b^ |
| Enzyme B | 60.28 | 29.68 | 1.137 | 1.32 | 0.100 | 0.433 | 0.224^abc^ | 0.016^ab^ | 0.000 | 0.0108^b^ | 0.124^b^ |
| Enzyme C | 45.24 | 32.90 | 1.576 | 6.86 | 0.146 | 0.905 | 0.400^a^ | 0.003^bc^ | 0.033 | 0.0227^a^ | 0.645^b^ |
| Enzyme D | 54.37 | 30.58 | 8.13 | 4.12 | 0.367 | 0.426 | 0.086^c^ | 0.001^c^ | 0.000 | 0.0177^a^ | 0.259^b^ |
| Enzyme E | 54.25 | 32.55 | 1.014 | 1.80 | 0.355 | 0.555 | 0.049^c^ | 0.003^bc^ | 0.000 | 0.0089^b^ | 0.184^a^ |
| SEM | 1.874 | 2.017 | 0.962 | 0.728 | 0.44 | 0.079 | 0.038 | 0.002 | 0.006 | 0.0027 | 0.055 |
| p value | 0.324 | 0.941 | 0.147 | 0.171 | 0.288 | 0.204 | 0.022 | 0.007 | 0.482 | 0.048 | 0.050 |

Enzyme A（1,500 U/g α-amylase）; Enzyme B（Enzyme A + 300 U/g [amylopectase](javascript:void(0);) +20,000 U/g [glucoamylase](javascript:void(0);)）; Enzyme C (Enzyme B + protease 10,000 U/g); Enzyme D (Enzyme C + 15,000 U/g xylanase); Enzyme E (Enzyme D + 200U/ g cellulase +1,000 U/g pectinase), respectively.

**S2 Table. Relative abundance of the dominant bacterial community in the cecal digesta of 23-day-old broiler chickens at family level**

|  | *Ruminococcaceae* | *Porphyromonadaceae* | *Veillonellaceae* | *Lactobacillaceae* | *Rikenellaceae* | *Bacteroidaceae* | *Acidaminococcaceae* | *Lachnospiraceae* | *Helicobacteraceae* | *Campylobacteraceae* | *Others* |
| --- | --- | --- | --- | --- | --- | --- | --- | --- | --- | --- | --- |
| Control | 17.10 | 15.68 | 3.974 | 4.282^ab^ | 10.14 | 6.069 | 10.904^a^ | 10.28 | 4.003 | 0.5578 | 17.01 |
| Enzyme A | 14.94 | 11.46 | 6.580 | 9.538^a^ | 5.68 | 8.680 | 7.705^ab^ | 9.40 | 3.138 | 1.6842 | 21.21 |
| Enzyme B | 20.46 | 14.45 | 5.887 | 9.434^a^ | 8.93 | 5.741 | 1.954^b^ | 11.45 | 5.164 | 3.3418 | 13.18 |
| Enzyme C | 16.79 | 12.28 | 4.735 | 3.025^ab^ | 10.14 | 8.342 | 2.517^b^ | 9.20 | 8.602 | 0.2010 | 24.16 |
| Enzyme D | 22.45 | 13.72 | 3.903 | 1.546^b^ | 10.00 | 6.760 | 6.838^ab^ | 14.06 | 3.215 | 0.8722 | 16.64 |
| Enzyme E | 22.22 | 8.41 | 9.178 | 0.982^b^ | 11.76 | 12.254 | 6.530^ab^ | 11.31 | 5.921 | 0.4937 | 10.93 |
| SEM | 0.0111 | 0.0130 | 0.01191 | 0.01079 | 0.0099 | 0.0088 | 0.00935 | 0.0059 | 0.00793 | 0.4683 | 0.01.53 |
| p value | 0.251 | 0.706 | 0.835 | 0.040 | 0.649 | 0.290 | 0.032 | 0.161 | 0.355 | 0.419 | 0.107 |

Enzyme A（1,500 U/g α-amylase）; Enzyme B（Enzyme A + 300 U/g [amylopectase](javascript:void(0);) +20,000 U/g [glucoamylase](javascript:void(0);)）; Enzyme C (Enzyme B + protease 10,000 U/g); Enzyme D (Enzyme C + 15,000 U/g xylanase); Enzyme E (Enzyme D + 200U/ g cellulase +1,000 U/g pectinase), respectively.

**S3 Table. Relative abundance of the dominant bacterial community in the cecal digesta of 23-day-old broiler chickens at genus level**

|  | *Barnesiella* | *Megamonas* | *Lactobacillus* | *Alistipes* | *Faecalibacterium* | *Bacteroides* | *Phascolarctobacterium* | *Helicobacter* | *Campylobacter* | *unidentified_Gastranaerophilales* | *Others* |
| --- | --- | --- | --- | --- | --- | --- | --- | --- | --- | --- | --- |
| Control | 13.75 | 3.929 | 4.281^ab^ | 9.786 | 6.089 | 6.069 | 0.904^a^ | 4.003 | 0.5558 | 1.086 | 39.55 |
| Enzyme A | 8.93 | 6.350 | 9.538^a^ | 5.551 | 6.412 | 8.680 | 7.705^ab^ | 3.138 | 1.6842 | 1.783 | 40.23 |
| Enzyme B | 13.26 | 5.859 | 9.434^a^ | 8.848 | 12.951 | 5.741 | 1.954^b^ | 5.164 | 3.3408 | 0.593 | 32.86 |
| Enzyme C | 09.64 | 4.733 | 3.024^ab^ | 10.052 | 5.847 | 8.342 | 2.517^b^ | 8.602 | 0.2010 | 2.847 | 44.19 |
| Enzyme D | 6.87 | 3.901 | 1.546^b^ | 9.878 | 10.426 | 6.760 | 6.838^ab^ | 3.215 | 0.8712 | 3.100 | 46.59 |
| Enzyme E | 5.67 | 9.178 | 0.982^b^ | 11.474 | 11.619 | 12.254 | 6.530^ab^ | 5.921 | 0.4937 | 0.927 | 34.95 |
| SEM | 0.0134 | 0.01177 | 0.01079 | 0.00976 | 0.01104 | 0.00879 | 0.00935 | 0.00793 | 0.004683 | 0.00392 | 0.0180 |
| p value | 0.443 | 0.831 | 0.040 | 0.665 | 0.238 | 0.290 | 0.032 | 0.355 | 0.419 | 0.326 | 0.213 |

Enzyme A（1500U/g α-amylase）; Enzyme B（Enzyme A+300U/g [amylopectase](javascript:void(0);) +20000U/g [glucoamylase](javascript:void(0);)）; Enzyme C (Enzyme B+protease 10000U/g); Enzyme D (Enzyme C + 15000U/g xylanase); Enzyme E(Enzyme D+ 200U/g Cellulase +1000U/g Pectinase), respectively


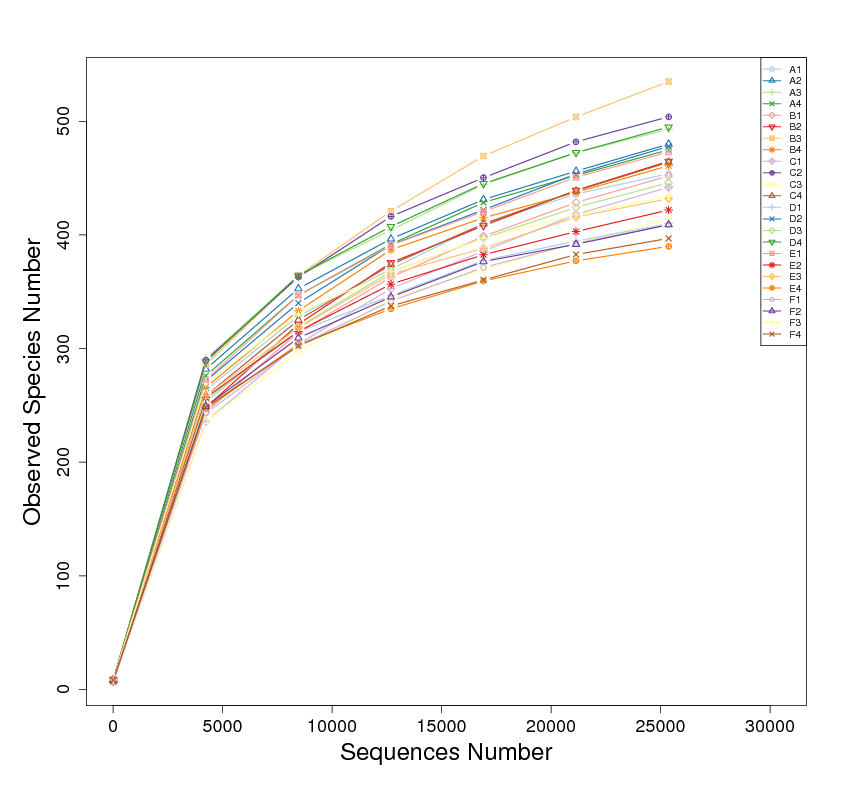


**S1 Fig. Rarefaction curves of samples clustered at 95% sequences identity.**

Sample information is list on the right side. A: the control; B: Enzyme A （1,500 U/g α-amylase; C: Enzyme B（Enzyme A + 300 U/g [amylopectase](javascript:void(0);) + 20,000 U/g [glucoamylase](javascript:void(0);)）; D: Enzyme C (Enzyme B+ 10,000 U/g protease); E: Enzyme D (Enzyme C + 15,000 U/g xylanase); F: Enzyme E (Enzyme D + 200 U/g cellulase + 1,000 U/g pectinase), respectively.


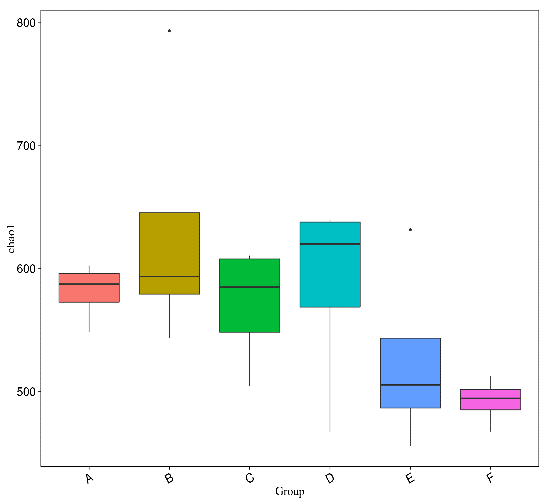

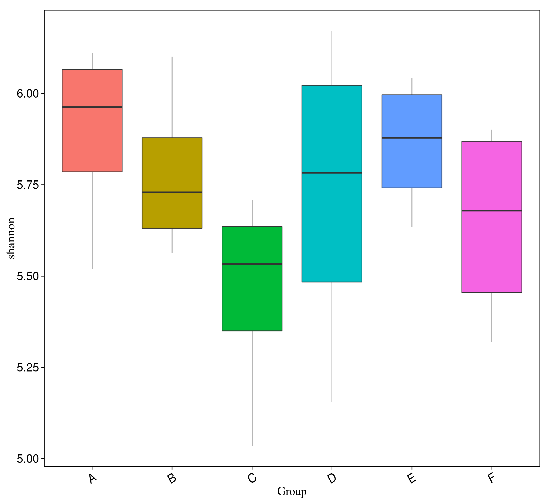

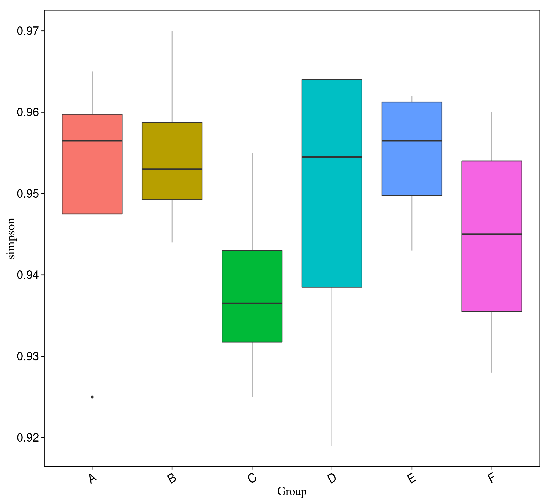


(a)

(b)

(c)

**S2 Fig. Richness and diversity of microbiome in cecal content. Chao 1 (a), Shannon diversity (b) and Simpson (c) indices are represented by box plots.**

Each box plot contains of means from four samples. The heavy black bars represent the median value**.** The bottom and top of the box, respectively, indicate the first and third quartiles. The black lines extend to the minimum and maximum values. A: the control; B: Enzyme A （1,500 U/g α-amylase; C: Enzyme B（Enzyme A + 300 U/g [amylopectase](javascript:void(0);) + 20,000 U/g [glucoamylase](javascript:void(0);)）; D: Enzyme C (Enzyme B+ 10,000 U/g protease); E: Enzyme D (Enzyme C + 15,000 U/g xylanase); F: Enzyme E (Enzyme D + 200 U/g cellulase + 1,000 U/g pectinase), respectively.


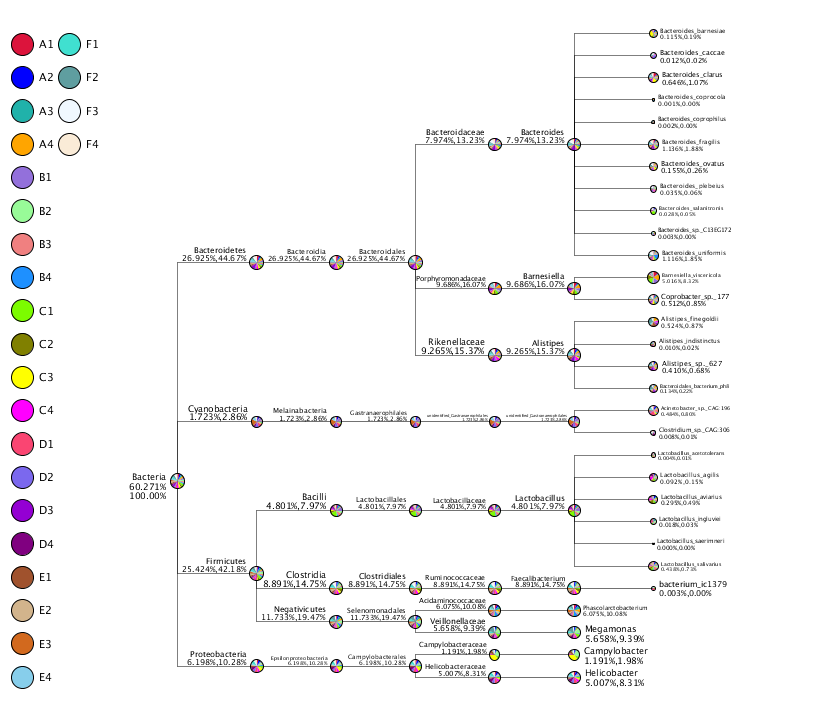


**S3 Fig. Detailed taxon tree**

A: the control; B: Enzyme A （1,500 U/g α-amylase; C: Enzyme B（Enzyme A + 300 U/g [amylopectase](javascript:void(0);) + 20,000 U/g [glucoamylase](javascript:void(0);)）; D: Enzyme C (Enzyme B+ 10,000 U/g protease); E: Enzyme D (Enzyme C + 15,000 U/g xylanase); F: Enzyme E (Enzyme D + 200 U/g cellulase + 1,000 U/g pectinase), respectively.
